# Characteristics of Functional Hyperthermia Detected in an Outpatient Clinic for Fever of Unknown Origin

**DOI:** 10.3390/jcm13030889

**Published:** 2024-02-03

**Authors:** Kosuke Oka, Kazuki Tokumasu, Hideharu Hagiya, Fumio Otsuka

**Affiliations:** 1 Department of General Medicine, Okayama University Graduate School of Medicine, Dentistry and Pharmaceutical Sciences, Okayama 700-8558, Japan; tokumasu@okayama-u.ac.jp (K.T.); fumiotsu@md.okayama-u.ac.jp (F.O.); 2 Department of Infectious Diseases, Okayama University Hospital, Okayama 700-8558, Japan; hagiya@okayama-u.ac.jp

**Keywords:** C-reactive protein (CRP), fever of unknown origin (FUO), functional hyperthermia (FH), psychiatric disorder, psychogenic fever

## Abstract

**Background:** Functional hyperthermia (FH) is characterized by hyperthermia resulting from sympathetic hyperactivity rather than inflammation, and it is frequently overlooked by medical practitioners due to the absence of abnormalities in a medical examination. Although FH is an important differential diagnosis for fever of unknown origin (FUO), the literature on FUO cases in Japan lacks information on FH. In this study, we aimed to uncover the population of FH patients hidden in FUO cases. **Methods:** An outpatient clinic for FUO was established at Okayama University Hospital, and 132 patients were examined during the period from May 2019 to February 2022. **Results:** A diagnosis of FH was made in 31.1% of the FUO cases, and FH predominantly affected individuals in their third and fourth decades of life with a higher incidence in females (68.3%). The frequency of a history of psychiatric illness was higher in patients with FH than in patients with other febrile illnesses. Although the C-reactive protein (CRP) is generally negative in FH cases, some obese patients, with a body mass index ≥ 25 had slightly elevated levels of CRP but were diagnosed with FH. **Conclusions:** The results showed the importance of identifying FH when encountering patients with FUO without any organic etiology.

## 1. Introduction

Functional hyperthermia (FH), also known as psychogenic fever, manifests as an elevation in body temperature that is not orchestrated by inflammatory cytokines but is a result of sympathetic hyperactivity [1,2]. Increased body temperature secondary to various types of stress has been studied in animal models, and various acute psychological stresses have been reported to increase body temperature [3,4,5,6,7,8,9,10,11,12,13,14,15,16,17,18,19,20]. Of interest, such a stress-induced increase in body temperature was also observed in many species of animals other than rats and mice [21,22,23,24,25,26,27,28,29,30]. Since the publication of a report in 1977 showing that prostaglandins act on the preoptic area of the hypothalamus to cause fever, the mechanism of the control of body temperature has been gradually unveiled [31]. It has been suggested that both inflammatory diseases and FH result in elevated body temperature due to increased heat production via adrenergic β3 receptors and due to the suppression of heat release by vasoconstriction via α1 receptors stimulated by the dorsomedial hypothalamus (DMH) [32,33].

In cases of infectious fever, temperature elevation is caused by the prostaglandin E2 deinhibition of inhibitory inputs from the hypothalamic preoptic area to the DMH [34,35,36]. On the other hand, in cases of FH, temperature elevation is caused during stress through input signals from the dorsal peduncular cortex/dorsal tenia tecta (DP/DTT) in the medial prefrontal cortex area [37,38], wherein stress signals from multiple brain regions can be accumulated and connected to the induction of stress responses. Hence, a single stress stimulus causes transient hyperthermia, and continued chronic exposure to a stressor can lead to a habitually elevated body temperature without exposure to the stressor [39,40,41].

In humans, FH occurs mainly in young individuals and adolescents, and it is characterized by an absence of elevated inflammatory markers and the ineffectiveness of non-steroidal anti-inflammatory drugs (NSAIDs) [42,43,44,45]. Individuals afflicted with FH frequently complain of fatigue, headache, insomnia, and other symptoms. Even a temperature marginally surpassing 37 °C becomes distressing because it exacerbates fatigue [46]. Since the main symptom of FH is prolonged fever, patients often visit a department of internal medicine for the diagnosis and treatment of fever of unknown origin (FUO). However, FH is inadequately recognized among internists, and it frequently escapes notice due to the dearth of specific clinical findings or biomarkers. The confirmation of diagnosis hinges on the elevation of body temperature in response to psychological stress or the normalization of body temperature after the removal of the stressor. Explicit diagnostic criteria for FH have not yet been established, and diagnosis depends on the subjective clinical discernment of the attending clinician.

In general, FUO is one of the most challenging medical conditions, with causative diseases exhibiting diversity and variance contingent upon regional characteristics and practice settings. Since Petersdorf and Beeson reported their seminal study on FUO in 1961, a plethora of investigations on FUO have been reported [47]. Concerning the epidemiology of FUO cases in Japan, prevailing reports suggest relatively high incidences of autoimmune and malignant diseases as causative factors, which are attributable to improved diagnostic capabilities and an aging population [48,49]. Goto’s study indicated the importance of encompassing a broad spectrum of febrile patients in studies for patients with temperatures surpassing 37 °C, challenging the conventional definition of FUO, which stipulates a fever of 38.3 °C or higher [50]. Previous studies, including our previous study, have shown the diagnostic utility of disease classification in febrile patients, the potential association between subclinical thyrotoxicosis and tachycardia during fever, and the efficacy of procalcitonin in febrile patients [51,52,53].

Although the significance of FH as a pivotal differential diagnosis in the domain of FUO has been proposed in several reports, there has been no study in which FH is incorporated into an investigation of the breakdown of final diagnoses in cases of FUO. Earlier reports suggest that approximately 20–30% of febrile cases remain undiagnosed, and we suspect that FH may account for some of these undiagnosed cases. We established an Outpatient Clinic for Fever of Unknown Origin at the Department of General Medicine of Okayama University Hospital. Through comprehensive analysis of FUO cases, we aimed to elucidate the prevalence of FH in cases of FUO, delineate the patient cohort for which the cause remained elusive in previous reports, and scrutinize trends and pathological conditions among patients presenting with FH.

## 2. Patients and Methods

### 2.1. Inclusion of Patients

Of the 146 patients who were referred to the Outpatient Clinic for FUO at Okayama University Hospital during the period from May 2019 to February 2022, 132 patients (47 males and 85 females) were included in this study after the exclusion of patients under 20 years of age. The Outpatient Clinic for FUO accepts patients with persistent temperatures of 37 °C or higher without being bound by the classic definition of FUO, for which the diagnosis is difficult. Their diagnostic breakdown and characteristics were systematically analyzed. The study protocol (#K-2308035) received approval from the Institutional Review Board (IRB) of Okayama University Graduate School of Medicine, Dentistry, and Pharmaceutical Sciences.

### 2.2. Definition of FH

In this study, the definition of FH was based on the following conditions according to previous reports [1,2,50]: (1) prolonged hyperthermia of 37 °C or higher for two months or longer, (2) the exclusion of organic febrile illness (through various tests, such as blood tests, endocrine tests, CT scans, etc.), (3) the history of a definitive stressor and the ensuing onset of prolonged fever, and (4) reproducible temperature increases with the stressor or return to normal temperature when the stressor is resolved. FH was diagnosed when criteria 1 + 2 + (3 and/or 4) were met.

### 2.3. Laboratory Examination

All the blood tests, including blood chemistry tests, were performed using an auto-analyzer system at the central laboratory of Okayama University Hospital for the differential diagnosis of FUO. The main system in our central laboratory was as follows: assays for complete blood count were performed using an electrical resistance method and a cyanmethemoglobin method using ADVIA2120 (Bayer AG, Leverkusen, Germany), and assays for serum CRP were performed using the latex agglutination turbidimetric immunoassay and BM8040 (JEOL, Tokyo, Japan). Assays for serum lactate dehydrogenase were also performed using BM8040. Assays for serum ferritin, plasma adrenocorticotropin, serum cortisol, serum-free thyroxin, and thyrotropin were performed using an electro-chemiluminescence immunoassay (ECLIA) and Cobas 8000 (F. Hoffmann-La Roche AG, Basel, Switzerland). 

Specifically, the assay for the serum soluble interleukin-2 receptor was an enzyme immunoassay (EIA) using LUMIPULSE L2400 (Fujirebio, Tokyo, Japan). The assay for the serum anti-nuclear antibody was performed using a fluorescent antibody method in the laboratory. The assay for serum rheumatoid factor was a latex immunoturbidimetric assay using BM6050 (JEOL). The assay for serum anti-cyclic citrullinated peptide antibodies was a chemiluminescent enzyme immunoassay (CLEIA) using ARCHITECT i2000SR (Abbott, Chicago, IL, USA). The assay for serum anti-neutrophil antibodies was a fluorescence enzyme immunoassay (FEIA) using ImmunoCAP250 Phadia (Thermo Fisher Scientific, Waltham, MA, USA). Assays for serum cytomegalovirus antibodies were FEIAs using VIDAS (bioMerieux, Craponne, France). The assay for Epstein–Barr (EB) viral nucleic acid quantification was performed by a real-time polymerase chain reaction in the laboratory.

### 2.4. Statistical Analysis

The Mann–Whitney U test, Kruskal–Wallis test, and Fisher’s exact probability test were used to analyze each group of data. In cases where there were discrepancies in the Kruskal–Wallis test, Steel–Dwass’s post hoc test was used to ascertain which means exhibited differentiation. All statistical analyses were performed using EZR version 4. 2. 2; a *p*-value less than 0.05 was deemed indicative of a statistically significant difference. EZR (Saitama Medical Center, Jichi Medical University, Saitama, Japan) was used as the interface for R (The R Foundation for Statistical Computing, Vienna, Austria). To be precise, it is a modified version of the R commander curated to integrate statistical functions frequently employed in biostatistics [54].

## 3. Results

### 3.1. Clinical Backgrounds of the Patients with FUO

Table 1 shows the clinical backgrounds of the patients. The median age of the patients was 44 years. The majority of the patients were between the ages of 20 and 59 years. The patients included 85 females (64.4%) and 47 males (35.6%). The median axillary body temperature of the patients at the initial visit was 36.9 °C. The median duration of fever before the first visit to our clinic was 63 days. The median body mass index (BMI) of the patients was 21.1. In addition, 43 patients (32.6%) had a history of smoking.

### 3.2. Breakdown of the Final Diagnosis of FUO

**Figure 1:**
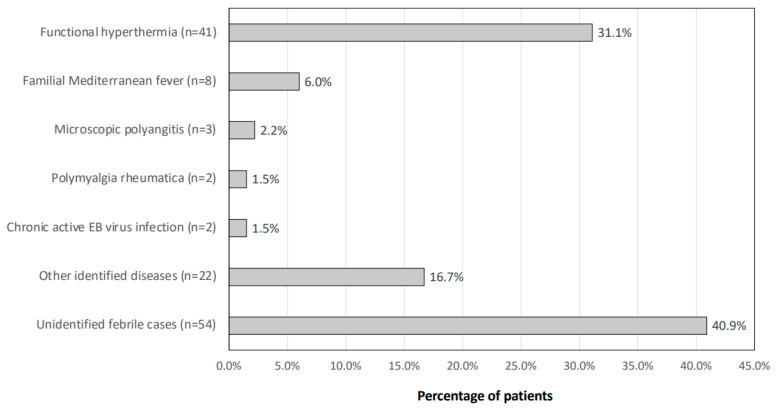
Final diagnosis of the patients with FUO. The diagnostic breakdown of patients who visited the Outpatient Clinic for Fever of Unknown Origin is shown.

Figure 1 shows a breakdown of the final diagnosis in individuals with FUO. A diagnosis of FH was made in 41 (31.1%) of the patients. There were 17 FH cases that met only the criteria of (3), 3 FH cases that met only the criteria of (4), and 21 FH cases that met the criteria of (3) and (4). Of the 41 patients with FH, 11 patients developed FH secondary to some physical event, such as an upper respiratory tract infection. Thirteen patients had joint pain, and four patients had skin rashes. Other diagnoses were familial Mediterranean fever in 8 patients (6.0%), microscopic polyangiitis in 3 patients (2.2%), polymyalgia rheumatica in 2 patients (1.5%), a chronic active EB virus infection in 2 patients (1.5%), and other identified diseases in 22 patients (16.7%). There were 54 unidentified febrile cases (40.9%). For the process of diagnosing FUO, 130 patients (85.6%) underwent a CT scan of their whole body, and 10 patients (7.6%) underwent a PET-CT scan. In addition, 5 patients received genetic testing in order to detect familial Mediterranean fever. As shown in Table 2, other identified diseases included myelodysplastic syndromes, adult-onset Still’s disease, miliary tuberculosis, HIV infection, rheumatoid arthritis, dermatomyositis, cytomegalovirus infection, Graves’ disease, and Fitzhugh–Curtis syndrome.

### 3.3. Distributions of Ages and Genders of Patients with FH

**Figure 2:**
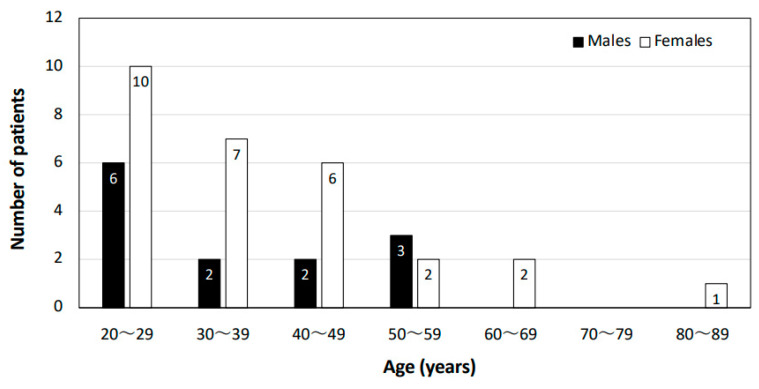
Characteristics of patients with FH. The age and gender distributions of patients diagnosed with FH in the Outpatient Clinic for Fever of Unknown Origin are shown.

Figure 2 shows the distribution of ages and genders in the patients diagnosed with FH. Patients with FH were predominantly women in their 20 s to 40 s. Males accounted for 31.7% of the cases, and females accounted for 68.3% of the cases.

### 3.4. Past Psychiatric Disorders in the Patients with FH

**Figure 3:**
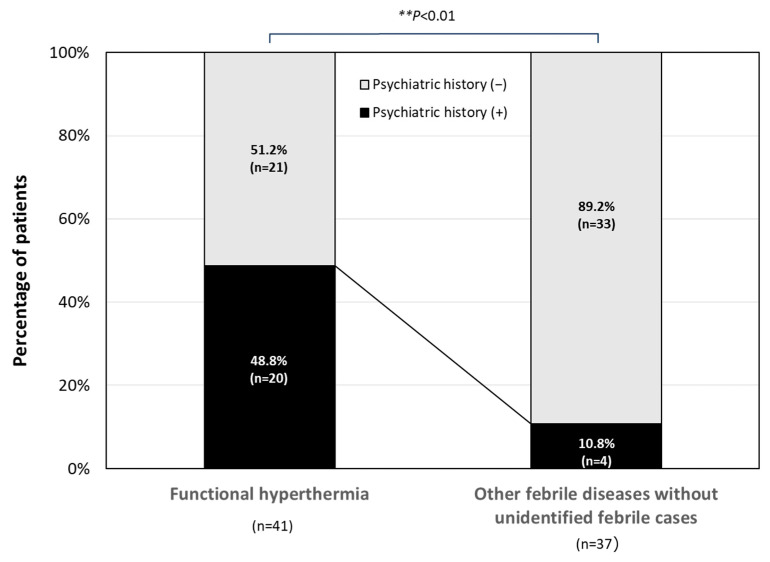
Past psychiatric disorders in patients with FH. The psychiatric histories of patients with FH are shown. Data were analyzed using Fisher’s exact probability test. ** *p* < 0.01 between the indicated groups.

Figure 3 shows the percentage of patients diagnosed with FH who previously attended a psychiatrist for any reason. FH patients with a psychiatric history accounted for about half (48.8%) of the FH patients, and this percentage was significantly higher than that in the organic disease group (10.8%; *p* < 0.01).

### 3.5. Interrelationship between Serum CRP Level and BMI in Patients with FH

**Figure 4:**
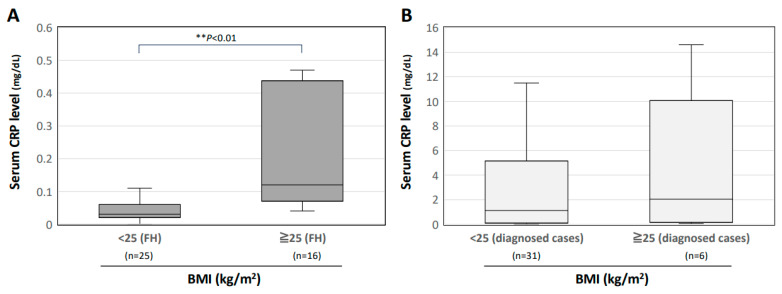
Relationships between the serum CRP level and obesity in patients with FH. Serum CRP levels are shown in two BMI groups (<25 and ≥25) (**A**) for patients with FH and (**B**) for patients with a diagnosis other than FH. The median is shown by the horizontal bar within the box, and the IQR is shown by the upper and lower horizontal bars of the box. The upper and lower horizontal bars outside the box represent the maximum and minimum values within 1.5 times the interquartile range. Data were analyzed using the Kruskal–Wallis test. If differences were detected, Steel–Dwass’s post hoc test was used to determine divergent means. ** *p* < 0.01 between the indicated groups.

Figure 4A shows the serum CRP levels in FH patients in two BMI-dependent groups, and Figure 4B shows the serum CRP levels in patients with a diagnosis other than FH in the two BMI-dependent groups. Obese patients with BMI ≥ 25 showed significantly higher serum CRP levels than those in patients with BMI < 25 (*p* < 0.01). There were no significant differences between the two BMI groups for patients with a diagnosis other than FH.

## 4. Discussion

In the present study, we show the proportion of FH cases in FUO cases and the clinical characteristics of FH. FH cases accounted for about 30% of the FUO cases examined in our outpatient clinic for FUO. Although there have been reports on cases of FUO in Japan, including reports from Okayama University Hospital, those reports predominantly focused on inflammatory diseases such as infectious diseases, malignant diseases, and collagen diseases with no mention of FH [46,48,49,51,53]. The reason why FH has not been described in previous reports on FUO may be that FH has been relegated to the unidentified category of fever cases because there are no abnormalities that can be identified in a medical examination, and its presence is relatively unfamiliar to physicians. However, our study suggests that FH is a crucial differential diagnosis and that FH cases account for the majority of FUO cases. 

The majority of patients with persistent FUO have a favorable prognosis, the characteristics of which align well with those of FH [50,55]. In cases of FUO examined in departments other than internal medicine, FH was reported to be diagnosed in 18.3%, 5.2%, and 4.4% of outpatients in pediatrics, child psychiatry, and obstetrics and gynecology departments, respectively [43]. Consequently, FH is not uncommon in clinical practice and is likely to be diagnosed in various medical departments. Internists, who play a pivotal role in fever treatment, need to be aware of FH.

In this study, familial Mediterranean fever was the second most prevalent cause of fever after FH. Familial Mediterranean fever, categorized as an autoinflammatory disease, belongs to a category that is different from infectious diseases, collagen diseases, and malignant diseases, which historically served as representative causes of FUO. It is imperative to adopt a flexible approach in the identification of the cause of FUO that is not subjected to constraints imposed by antecedent reports. The causative diseases of FUO other than FH and the familial Mediterranean fever in the present study were similar to those previously reported. Patients diagnosed with FH were predominantly women in their 20 s to 40 s, with a male-to-female ratio of 1:2.15. Although patients aged 19 years or younger were excluded from the present study, making direct comparisons with other studies difficult, the results of this study suggest that FH occurs more frequently in relatively young women, in accordance with the results of previous studies.

The results of the present study also suggest that patients with a history of psychiatric consultation are a high-risk group for FH. Since FH is characterized by an elevation in body temperature under stressful mental conditions, a history of psychiatric visits may be helpful in diagnosing FH in febrile patients without organic disease. It is also considered to be important to resolve the backgrounds of psychiatric disorders for the treatment of patients with FH [56]. In the present study, FH in approximately one-quarter of the patients with FH was possibly triggered by their preceding physical stress. Chronic fatigue syndrome (CFS), similarly triggered by physical stress events, is known to be complicated by hyperthermia [1,57,58,59]. The similarity between the modes of onset of FH and CFS and the fact that FH and CFS can merge with each other suggest that FH and CFS may have a common pathogenesis [60]. Further investigation is needed to determine whether patients with FH concurrently manifest CFS.

In the FH group, obese patients with a BMI ≥ 25 kg/m^2^ showed a significantly higher CRP level than that in patients with a BMI < 25 kg/m^2^. Previous studies showed that there is a marginal elevation of CRP in obese patients, and a similar trend was observed in the present study [61,62,63]. While CRP is generally negative in patients without inflammatory disease, including those with FH, obese patients may show a slightly heightened inflammatory response even in the absence of inflammatory disease. Consequently, in obese patients with FUO, FH should remain within the differential diagnosis, even if they have a slightly increased level of CRP. 

The present study has several limitations. This study was a single-center study and only included patients who visited the Outpatient Clinic for Fever of Unknown Origin, not a general outpatient clinic. The high prevalence of FH in comparison with the prevalence of infectious diseases, collagen diseases, and malignant diseases, historically reported as the primary pathogenesis of FUO, may be due to the single-center nature of this study, in which only patients visiting an outpatient clinic for FUO were included. Correspondingly, data from another outpatient clinic for FUO in the National Center for Global Health and Medicine indicated that FH accounted for 23.5% of FUO cases [64]. These findings imply that the high prevalence of FH in the FUO cases in this study, unlike in previous studies, may merely indicate that FH is more likely to be encountered in an outpatient clinic for FUO. 

In conclusion, this study shows that FH may account for a substantial proportion of FUO cases and that FH occurs predominantly in women in their 20 s to 40 s. This study also shows that patients with a history of psychiatric visits are at high risk for FH and that the possibility of FH cannot be excluded even if serum CRP levels are slightly elevated in FUO cases among obese patients. Recognizing that FH is an important differential disease in FUO practice could help medical practitioners to accurately diagnose and guide FUO patients to a resolution.

## Figures and Tables

**Table 1:** Background of patients who visited the Outpatient Clinic for Fever of Unknown Origin.

Patients’ Background		
Age	Case Number (Total 132 Patients)	Male/Female
Median [IQR], years	44 [28–61.5]	47/85
20–29 years	42 (31.8%)	16/26
30–39 years	16 (12.1%)	4/12
40–49 years	20 (15.2%)	5/15
50–59 years	18 (13.6%)	9/9
60–69 years	11 (8.3%)	3/8
70–79 years	16 (12.1%)	7/9
80–89 years	9 (6.8%)	3/6
**Body temperature**		
Median [IQR] (°C)	36.9 [36.6–37.2]	
**Fever duration**		
Median [IQR] (days)	63 [30–240]	
**BMI**		
Median [IQR] (kg/m^2^)	21.1 [18.5–26.1]	

IQR: Interquartile range.

**Table 2:** List of final diagnoses included in other identified diseases.

Final Diagnosis
Adult-onset Still’s disease
Aseptic meningitis
Aspiration pneumonia
Carcinomatous pleurisy
Clostridium difficile-associated diarrhea
Cytomegalovirus infection
Dermatomyositis
Drug allergy
Fitz–Hugh–Curtis syndrome
Graves’ disease
Human immunodeficiency virus infection
Inflammatory lymphadenitis
Lung abscess
Miliary tuberculosis
Myelodysplastic syndromes
Neurogenic fever
PFAPA syndrome
Pharyngeal carcinoma
Post-vaccination reaction to coronavirus vaccine
Pseudogout
Rheumatoid arthritis
Ureteral obstruction

## Data Availability

Detailed data are available if requested from the corresponding author.

## Abbreviations

Chronic fatigue syndrome (CFS), C-reactive protein (CRP), Epstein-Barr (EB), fever of unknown origin (FUO), functional hyperthermia (FH), and non-steroidal anti-inflammatory drugs (NSAIDs).
